# Disability and functioning in primary and secondary hip osteoarthritis in Benin

**DOI:** 10.4102/ajod.v9i0.675

**Published:** 2020-11-12

**Authors:** Todègnon F. Assogba, Didier D. Niama-Natta, Toussaint G. Kpadonou, Teefany Lawson, Philippe Mahaudens, Christine Detrembleur

**Affiliations:** 1 Neuro Musculo Skeletal Lab (NMSK), Faculté des Sciences de la Motricité, Université Catholique de Louvain, Brussels, Belgium; 2 Clinique Universitaire de Médecine Physique et de Réadaptation, Centre National Hospitalier et Universitaire Hubert K. Maga, Cotonou, Benin; 3 Service d’Orthopédie et de Traumatologie de l’Appareil Locomoteur, Cliniques Universitaires Saint-Luc, Brussels, Belgium

**Keywords:** hip osteoarthritis, pain, gait speed, quality of life, Africa

## Abstract

**Background:**

In Africa, primary hip osteoarthritis seems to be less frequent than in Europe. Sickle cell disease is responsible for aseptic osteonecrosis of the femoral head associated with secondary hip osteoarthritis. Very little evidence is available on the influence of aetiology (primary and secondary) and radiographic status on pain and disability in a Beninese population with hip osteoarthritis.

**Objectives:**

The aim of this study was to compare the impacts of aetiology and radiographic status on pain, disability and quality of life in a Beninese population with hip osteoarthritis.

**Method:**

This was a descriptive cross-sectional study, including participants recruited in the Clinic of Physical Medicine and Rehabilitation at the National Teaching Hospital in Cotonou.

Assessment was based on the International Classification of Functioning, Disability and Health model. The main outcomes were severity of osteoarthritis, pain, range of motion, muscle strength, gait speed and quality of life. Statistical comparisons between the aetiologies were performed using a *t*-test or rank sum test. One-way analysis of variance was used to test the effect of radiographic status.

**Results:**

Forty-nine participants (26 women and 23 men; mean age [standard deviation] 40.5 [17.9] years) were recruited. According to the aetiology (59.2% and 40.8% of primary and secondary osteoarthritis, respectively), there were no significant differences for any of the outcomes. Grades I, II, III and IV osteoarthritis were observed in 22.4%, 14.3%, 26.5% and 36.7% of the participants, respectively. Participants with grade IV osteoarthritis were more affected than those with grades I, II and III based on the Kellgren and Lawrence classification.

**Conclusion:**

Aetiology did not influence pain, gait speed or quality of life. Participants with grade IV osteoarthritis had more pain, were more limited in walking and had a more impaired quality of life.

## Introduction

Osteoarthritis is a chronic condition that affects 11% of the general adult population and is the most common form of arthritis (Pereira et al. 2011). Hip osteoarthritis (HOA) is among the most common joint diseases and is therefore a major social and health problem (Danielsson & Lindberg 1997). Hip osteoarthritis is one of the main causes of disability in the elderly (Loeser 2010), with prevalence increasing progressively with age (Jordan et al. 2009; Oliveria et al. 1995).

However, little is known about the prevalence of HOA in Africa. As demonstrated by Dowman et al. (2012), HOA is often seen as a minor health problem and has been neglected in research and resource allocation throughout Africa despite potential related disabilities (such as abnormal gait patterns and lower physical function) and decreased quality of life (QoL).

According to their aetiologies, there are two types of HOA: primary and secondary. The primary HOA is idiopathic with no hip malformation. It is the most common HOA in Western countries. The development of primary HOA is favoured by a probable genetic predisposition that affects cartilage metabolism, with other contributing factors such as biomechanical constraints (Englund 2010). Secondary HOA is a result of changes in the microenvironment of the cartilage. Its aetiologies include congenital hip abnormalities, metabolic defects, infections and blood disorders such as sickle cell disease (Kpadonou et al. 2011; Oniankitan et al. 2009; Ouedraogo et al. 2015). This hereditary disease is more frequent in sub-Saharan Africa, India, the Middle East and Brazil (Lespasio, Sodhi & Mont 2019). Sickle cell disease is responsible for aseptic osteonecrosis of the femoral head resulting from ischemia associated with secondary HOA (Akinyoola, Adediran & Asaleye 2007; Kpadonou et al. 2011; Oniankitan et al. 2009; Ouedraogo et al. 2015).

Several factors that would contribute to disability and pain caused by HOA have been identified (Arokoski et al. 2004; Juhakoski et al. 2008; Kondo et al. 2017; Steultjens et al. 2000; Thumboo, Chew & Lewin-Koh 2002). According to Dekker et al. (1992):

[*R*]adiographically assessed degeneration of cartilage and bone is associated with pain and disability, but it appears that the association is rather weak Thus, it seems that status of the joint is not enough to explain pain and disability in patients with osteoarthritis.

Arokoski et al. (2004) have shown that men with HOA have significantly lower muscle strength than their age- and sex-matched controls. Van Baar et al. (1998) showed that in HOA, disability was associated with muscle strength, joint range of motion (ROM), pain, ability to cope with pain and psychological well-being, but the level of cartilage and bone degradation assessed by radiography did not influence the disability.

In accordance with the literature, there are few studies in Africa, and particularly in the Republic of Benin, that have examined factors associated with functioning and disabilities in patients with HOA. In sub-Saharan Africa, secondary HOA occurs in a younger population, and aseptic osteonecrosis of the femoral head is one of the most reported risk factors (Kpadonou et al. 2011; Oniankitan et al. 2009; Ouedraogo et al. 2015).

The main purpose of this study was to compare the impact of primary and secondary HOA on body structures and function, activity and QoL. The secondary purpose was to explore the impact of radiographic state in modifying body structures, function and disability in Beninese participants with HOA. We hypothesised that Beninese participants with secondary HOA would be more affected with regard to impairments, activity limitations and QoL than those with primary HOA. We further hypothesised that greater degradation of cartilage and bones, assessed by X-ray, would be associated with more impairments, activity limitations and reduced QoL.

## Materials and method

### Study design and setting

This is a cross-sectional, descriptive study. Participants were recruited from a rehabilitation clinic and assessed once through a series of tests and questionnaires.

### Participants

This study focused on participants with HOA in the Republic of Benin, a West African country.

Participants were recruited from the Clinic of Physical Medicine and Rehabilitation at the National Teaching Hospital in Cotonou. Participants with bilateral or unilateral symptomatic HOA diagnosed by radiography were recruited. In participants with bilateral HOA, the most painful hip was selected for analysis. The diagnosis of HOA was based on the American College of Rheumatology classification criteria and/or Kellgren and Lawrence (1957) radiographic classification (Vignon et al. 1999). Participants with total hip replacements, neurological or psychiatric disorders or any deficit that would prevent them from completing the questionnaires and assessments were excluded.

### Recruitment procedure

Participants with an HOA diagnosis were initially identified from the rehabilitation care registry, focusing on the period between 01 June 2008 and 30 April 2019. From a contact list of 147 eligible participants, 69 were reached and invited to participate in the study. Out of the 69 reached, 4 died (information given by family members), 11 had total hip replacements and 2 refused to participate. The remaining 52 participants who agreed to participate were invited for a physical medicine consultation. After the screening consultation, three participants were excluded because they did not meet the selection criteria (diagnosis not consistent with HOA).

Finally, 49 participants meeting the inclusion criteria participated in the study.

### Outcomes

**FIGURE 1:**
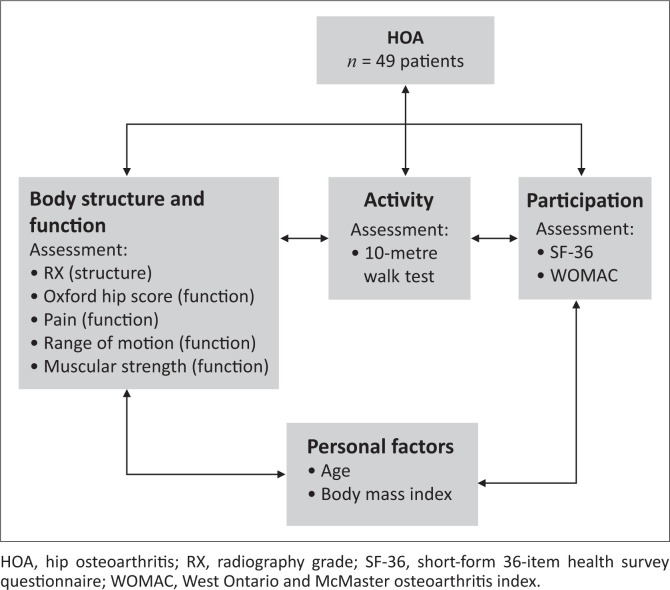
Evaluation of patients using the International Classification of Functioning, Disability and Health model.

Assessments were based on the International Classification of Functioning, Disability and Health (ICF) model (WHO 2001). Applying this model to HOA will enable identification of the impairments, activity limitations and participation restrictions experienced by the participants (Figure 1).

### Measurements of International Classification of Functioning, Disability and Health body structures and function

The state of cartilage and bone was assessed by radiography with the Kellgren and Lawrence (1957) radiographic classification (Vignon et al. 1999). Pain was assessed with the Oxford hip score (OHS) and the numerical rating scale (NRS), hip ROM with goniometer and hip strength with the Medical Research Council (MRC) scale.

The OHS is a standard patient-reported outcome measure, developed to assess function and pain in participants with HOA (Martinelli et al. 2011) or undergoing total hip replacement surgery, particularly in the context of clinical trials (Dawson et al. 1996; Murray et al. 2007).

The OHS has also been used for the assessment of participant outcomes, including physical therapy and use of joint supplements (Murray et al. 2007). The scale consists of 12 items with 5 categories of response, 0–4 (worst to best), with the overall score ranging from 0 to 48, where 48 represents the best score (Murray et al. 2007). The NRS is a unidimensional measure of pain intensity in adults, including those with chronic pain resulting from rheumatic diseases. The 11-point numeric scale ranges from 0, representing no pain, to 10, representing the most intense imaginable pain.

Hip ROM was measured by the goniometer, whose validity has been established (Prather et al. 2010). Hip flexion, adduction and abduction motions were measured in the supine position, with the opposite thigh fixed in a neutral position. Internal and external rotation of the hip was measured in prone (with the hip in a neutral position and the knee in 90° flexion) and sit position (with the hip and knee in 90° flexion). Hip extension was measured with the participant lying on one side (hip in a neutral position and knee in extension). Goniometer-based measure has shown high intrarater reliability (intraclass correlation = 0.82–0.95) except for adduction (ICC = 0.50–0.72) (Holm et al. 2000).

Hip muscle strength was measured using the MRC scale with a rating of 0–5. Psychometric studies of MRC have shown high reliability for osteoarthritis (intraclass coefficient, > 0.95) (Youdas, Madson & Hollman 2010). Participants were placed in a position that enabled support for their body, so that they could focus their efforts on the tested muscle. The tested muscle was first placed in an antigravity position. If muscles were too weak to move the segment against gravity, they were tested in the horizontal plane. The proximal part of the tested limb was stabilised to reduce the risk of compensation by other muscles than those tested. Manual resistance was applied in the plane of movement for the tested muscles. Resistance was applied by progressive pressure. In most cases, there was a long lever in the arm (Appendix 1).

### Measurements of International Classification of Functioning, Disability and Health activity

Activity limitations were assessed by the 10-metre walk test (10MWT). The test was performed on a 14-metre pathway, where each participant was asked to walk at his or her own comfortable speed. The participant’s performance was timed over the middle 10-metre course. The initial 2 metre served to reach a constant speed. The final 2 metre prevented the participant from decelerating prematurely (Juhakoski et al. 2008; Tani et al. 2018) (Appendix 2).

### Measurements of International Classification of Functioning, Disability and Health participation

To assess the QoL, the short-form 36-item (SF-36) health survey questionnaire and West Ontario and McMaster (WOMAC) osteoarthritis index (Appendices 3 and 4) were used. The SF-36 is a generic validated questionnaire, acceptable for the long-term measurements of QoL in osteoarthritis and validated in French (Hayes et al. 1995; Walters, Munro & Brazier 2001). This measure allows quantification of the physical and mental components (PC and MC) of QoL. It was shown to be reliable and valid (McHorney et al. 1994; McHorney, Ware & Raczek 1993) and consists of eight subscales: physical function, role limitations resulting from physical problems, bodily pain, general health perceptions, role limitations resulting from emotional problems, mental health, social functioning and vitality. Each score ranges from 0 to 100, where 0 is the poorest possible health state and 100 is the best health state.

The WOMAC questionnaire was developed to assess pain, joint stiffness and disability related to osteoarthritis of the knee and hip (Bellamy et al. 1988). It contains 24 questions, 5 related to pain, 2 to stiffness and 17 to physical function. It provides an excellent overview of a participant’s functional capacity and complements the more objective data provided by magnetic resonance imaging, arthroscopy, cartilage biopsy and radiographs.

### Data analysis

All variables with a normal distribution and equality of variance were presented as means with standard deviation (SD). For non-normally distributed variables, non-parametric descriptive statistics were used, and the results were expressed as medians and 1st and 3rd quartiles (25% – 75%). Statistical analyses were performed using SigmaPlot from SPSS (version 13.0). The significance level was set at *p* < 0.05.

*T*-test or Mann–Whitney test for non-parametric variables was used to determine if participants with secondary HOA are more affected in structure and function, activity and participation than those with primary osteoarthritis. To establish if the degree of degradation in the Kellgren and Lawrence radiographic classification was associated with impairments, activity limitations and decreased QoL, we used a one-way analysis of variance (ANOVA) if the normality and equality of variance tests passed or Kruskal–Wallis one-way ANOVA on ranks (if the normality and equality of variance tests failed) with one factor (factor = grade, that is, grade I, grade II, grade III and grade IV). A post hoc test was used to identify significant differences between the different radiographic grades.

### Ethical consideration

Adult participants or parents of minor participants freely gave signed consent to participate in the study. The clinical study was conducted with authorisation from the hospital institution and informed consent of the patients. This is the current regulation in Republic of Benin. This study is part of a thesis project that has received the approval of the local ethics committee of the University Clinic of Physical Medicine and Rehabilitation of the Centre National Hospitalier et Universitaire Hubert K. Maga in Cotonou/Benin under the number: CE 07-2018/MS/CNHU-HKM/CUMPR/CE/SP.

## Results

### Characteristics of participants

Our sample of 49 participants comprised 26 women and 23 men, with a mean age of 40.5 (SD 17.9) years (range, 11–70 years). Participants had an average body mass index (BMI) of 25.6 kg/m^2^ (SD 7.4); 40 participants had unilateral and 9 had bilateral HOA. According to Kellgren–Lawrence grades, the radiographic severity was of grade I in 22.5% (*n* = 11) of participants, grade II in 14.3% (*n* = 7), grade III in 26.5% (*n* = 13) and grade IV in 36.7% (*n* = 18).

Twenty (40.8%) participants had secondary osteoarthritis resulting from aseptic osteonecrosis of the femoral head, and 29 (59.2%) had primary osteoarthritis (Table 1).

**TABLE 1:** Characteristics of participants.

Characteristic	Value
**Gender ( % )**	
Male	53.1
Female	46.9
**Age (years), mean (SD)**	40.5 (17.9)
**Weight (kg), mean (SD)**	69.7 (20.8)
**Height (cm), mean (SD)**	163.7 (11.5)
**BMI (kg/m^2^), mean (SD)**	25.6 (7.3)
**Kellgren– Lawrence radiographic grade ( % )**	
I	22.5
II	14.3
III	26.5
IV	36.7
**Aetiology (%)**	
Primary	59.2
Secondary	40.8
**Affected hip (%)**	
Unilateral	81.6
Bilateral	18.4

BMI, body mass index; SD, standard deviation.

### Effects of aetiology on body structure and function, activity and participation

Participants with primary HOA were significantly older (median 51 [interquartile range – IQR – 43.5–61] vs. 23 [IQR 16–27.7] years, *p* < 0.001) and had significantly higher BMI (median 26.2 [IQR 24.7–31.9] vs. 21.5 [16.7–22.7] kg/m^2^, *p* < 0.001) than those with secondary HOA.

There were no statistically significant differences between primary and secondary HOA for pain assessed by NRS scale (mean 6.4 [SD 1.8] vs. 5.8 [SD 2.4], respectively, *p* = 0.35) and OHS scale (mean 23.6 [SD 8.1] vs. 26.4 [SD 9.9], *p* = 0.28). There were no statistically significant differences for hip ROM and muscles strength except for the external rotators (external rotator muscles 3 and 4/5 for secondary vs. primary HOA, respectively; *p* = 0.01) (Table 2).

**TABLE 2:** Comparison of body structures and function in primary versus secondary osteoarthritis.

Variables	Secondary osteoarthritis (*n* = 20)		Primary osteoarthritis (*n* = 29)		*p*
	Mean (SD)	Median [25% – 75%]	Mean (SD)	Median [25% – 75%]	
NRS (out of 10)	5.85	2.43	6.43	1.85	0.35
OHS (out of 48)	26.45	9.98	23.62	8.13	0.28
Flexion (°)	97.56	25.12	101.86	18.46	0.49
Extension (°)	19.11	9.56	19.93	9.04	0.76
Abduction (°)	23.56	12.59	25.5	13.78	0.62
Adduction (°)	18.5	9.96	19.67	8.44	0.66
Internal rotation (°)	25.41	10.16	23.62	9.37	0.53
External rotation (°)	25.77	9.97	21.96	11.34	0.23
Flexor muscles (out of 5)	3	3–4	3	3–4	0.90
Extensor muscles (out of 5)	3	3–4	3	3–4	0.78
Abductor muscles (out of 5)	3	3–4	4	3–4	0.18
Adductor muscles (out of 5)	3	3–3	3	3–4	0.38
Internal rotator muscles (out of 5)	3	3–4	4	3–4	0.31
External rotator muscles (out of 5)	3	3–3	4	3–4	0.01

NRS, numerical rating scale; OHS, Oxford hip score; SD, standard deviation.

In terms of activity (10MWT), no statistically significant differences were observed between participants with primary or secondary HOA (12.2 s vs. 12.7 s) (Table 3).

**TABLE 3:** Comparison of activity and participation in primary versus secondary osteoarthritis.

Variables	Secondary osteoarthritis (*n* = 20)		Primary osteoarthritis (*n* = 29)		*p*
	Mean	SD	Mean	SD	
10MWT (seconds)	12.72	5.87	12.19	5.58	0.75
SF-36 PC (%)	48.5	23.71	40.99	16.66	0.20
SF-36 MC (%)	61.28	25.29	56.34	23.99	0.50
WOMAC (%)	40.41	20.91	47.68	15.45	0.17

10MWT, 10-metre walk test; SD, standard deviation; SF-36 PC, short-form 36-item health survey questionnaire, physical component; SF-36 MC, short-form 36-item health survey questionnaire, mental component; WOMAC, West Ontario and McMaster osteoarthritis index.

In terms of QoL, the difference was not significant (*p* = 0.2, 0.5, 0.17) between primary and secondary aetiology (SF-36 PC, 40.9% vs. 48.5%; SF-36 MC, 56.8% vs. 61.2%; WOMAC, 47.6% vs. 40.4%) (Table 3).

### Effects of grades of X-rays on structure and function, activity and participation

Grade of HOA significantly affected function, activity and QoL (Tables 5 and 6). No statistically significant differences for age, weight, height, BMI, hip ROM or muscle strength were reported between grades I, II, III and IV of HOA (Tables 4 and 5).

**TABLE 4:** Comparison of age and anthropometry based on grades of hip osteoarthritis.

Variables	Grade I (*n* = 11)				Grade II (*n* = 7)				Grade III (*n* = 13)				Grade IV (*n* = 18)				*p*
	Mean	SD	Median	IQR [25% – 75%]	Mean	SD	Median	IQR [25% – 75%]	Mean	SD	Median	IQR [25% – 75%]	Mean	SD	Median	IQR [25% – 75%]	
Age (years)	-	-	42	21–55	-	-	29	16–46	-	-	38	20–55	-	-	46.5	28.5–62.75	0.58
Weight (kg)	62.86	16.33	-	-	65.29	16.48	-	-	76.84	30.09	-	-	69.64	14.76	-	-	0.41
Height (cm)	-	-	164	158–170	-	-	163	160–172	-	-	167.5	153.5–174.5	-	-	164.5	158.5–170.75	0.93
BMI (kg/m^2^)	-	-	24	19.1–25.1	-	-	23.3	19.1–25.8	-	-	25.5	17.3–38.1	-	-	26	22.6–28.5	0.47

BMI, body mass index; SD standard deviation; IQR, interquartile range

**TABLE 5:** Comparison of body structures and function based on grades of hip osteoarthritis.

Variables	Grade I (*n* = 11)				Grade II (*n* = 7)				Grade III (*n* = 13)				Grade IV (*n* = 18)				*p*
	Mean	SD	Median	IQR[25% – 75%]	Mean	SD	Median	IQR[25% – 75%]	Mean	SD	Median	IQR[25% – 75%]	Mean	SD	Median	IQR[25% – 75%]	
NRS (out of 10)	-	-	4	3–7	-	-	5	2.75–7.25	-	-	6	5–8	-	-	7	6–9	**0.02**
OHS (out of 48)	-	-	33	29–37	-	-	33	29–35	--	-	24	20.5–27.5	-	-	19	8.75–27	**˂ 0.001**
Flexion (°)	108.82	9.43	-	-	103.43	12.95	-	-	101	20.69	-	-	91.6	28.15	-	-	0.21
Extension (°)	20.6	7.25	-		19.57	10.5	-	-	19.54	10.42	-	-	19	9.39	-	-	0.98
Abduction (°)	-	-	30	20–44			22	20–40	-	-	20	17.5–34	-	-	10	10–32	0.11
Adduction (°)	-	-	20.7	9.41	18.83	9.68	-	-	18.91	8.88	-	-	18.47	9.46	-	-	0.95
Internal rotation (°)	29.54	10.03	-	-	20.33	12.09	-	-	22.75	10.06	-	-	23.29	6.72	-	-	0.19
External rotation (°)	-	-	30	20–38	-	-	19	11.5–32.5	-	-	16	12–26	-	-	20	15.75–30.5	0.19
Flexor muscles (out of 5)	-	-	4	3–4	-	-	4	3–4	-	-	3	2.5–4	-	-	3	3–3.25	0.24
Extensor muscles (out of 5)	-	-	3.5	3–4	-	-	3	3–3	-	-	3	3–3.75	-	-	3	3–4	0.29
Abductor muscles (out of 5)	-	-	4	3–4	-	-	4	3–4	-	-	3	3–4	-	-	3	3–4	0.31
Adductor muscles (out of 5)	-	-	3	3–3	-	-	4	3–4	-	-	3	3–3	-	-	3	3–3	0.15
Internal rotator muscles (out of 5)	-	-	4	3–4	-	-	3.5	3–4	-	-	4	3–4	-	-	3	2.75–4	0.36
External rotator muscles (out of 5)	-	-	3	3–4	-	-	3.5	3–4	-	-	3	3–4	-	-	3	3–4	0.52

NRS, numerical rating scale; OHS, Oxford hip score; SD, standard deviation; IQR, interquartile range.

Post hoc analysis NRS (Dunn’s method): Grade I ≠ Grade IV (*p* = 0.02).

Post-analysis OHS (Dunn’s method): Grade II ≠ Grade IV; I ≠ IV (*pw* < 0.001).

**TABLE 6:** Comparison of activity and participation based on grades of hip osteoarthritis.

Variables			Grade I (*n* = 11)				Grade II (*n* = 7)				Grade III (*n* = 13)				Grade IV (*n* = 18)		*p*
			
	Mean	SD	Median	IQR [25% – 75%]	Mean	SD	Median	IQR[25% – 75%]	Mean	SD	Median	IQR [25% – 75%]	Mean	SD	Median	IQR [25% – 75%]	
10MWT (seconds)	-	-	9.95	7.33–11	-	-	10.27	9.4–12.08	-	-	10.33	9.33–10.85	-	-	12.34	10.54–16.76	**0.008**
SF-36 PC (%)	59.43	13.44	-	-	55.18	19.18	-	-	46.39	11.85	-	-	28.64	18.16	-	-	**˂ 0.001**
SF-36 MC (%)	76.11	12.95	-	-	77.71	19.94	-	-	56.28	19.48	-	-	41.48	22.58	-	-	**˂ 0.001**
WOMAC (%)	-	-	30.2	12.5–40.62	-	-	37.5	29.7–44.3	-	-	42.18	38.8–53.64	-	-	57.29	46.35–73.96	**˂ 0.001**

SD, standard deviation; 10 MWT, 10 metre walk test; SF-36 PC, short-form 36-item health survey questionnaire, physical component; SF-36 MC, short-form 36-item health survey questionnaire, mental component; IQR, interquartile range.

Post hoc analysis 10MWT (Dunn’s method): Grade IV ≠ grade I (*p* = 0.01).

Post hoc analysis SF-36 PC (Holm Sidak method): Grade I ≠ grade IV (*p* < 0.001); Grade II ≠ grade IV (*p* = 0.002); Grade III ≠ grade IV (*p* = 0.014).

Post hoc analysis SF-36 MC (Holm Sidak method): Grade I ≠ grade IV (*p* < 0.001); Grade II ≠ grade IV (*p* < 0.001).

Post hoc analysis WOMAC (Dunn’s method): Grade I ≠ grade IV (*p* < 0.001).

Regarding pain, participants with grade IV HOA reported more pain (NRS, median 7 [IQR 6–9], *p* = 0.02; OHS, median 19 [IQR 8.7–27], *p <* 0.001) than those with grades I, II and III. Post hoc analysis revealed for NRS a difference between participants with grades I and IV (*p* = 0.02); for OHS there was a significant difference between participants with grades II and IV HOA on the one hand and participants with grades I and IV HOA on the other hand (*p* < 0.001) (Table 5).

Regarding hip ROM and muscle strength, there were no differences between any of the groups (Table 5).

With respect to activity, a significant statistical difference was also observed. Participants’ scores on the 10MWT were 9.9, 10.2, 10.3 and 12.3 s for grades I, II, III and IV HOA, respectively. Post hoc analysis revealed a significant difference between participants with grades I and IV HOA (*p* = 0.001) (Table 6).

A significant difference was observed for QoL with the WOMAC, SF-36 PC and SF-36 MC (*p* < 0.001 for all three outcomes). Post hoc analysis revealed for WOMAC a significant difference between participants with grades I and IV HOA (median 30.2 [IQR 12.5–40.6] vs. 57.29 [IQR 46.3–73.9], *p* < 0.001). Post hoc analysis revealed a significant difference between participants with grades I and IV, II and IV, III and IV HOA for the SF-36 PC (mean [SD]: 59.4 [13.4], 55.2 [19.2], 46.4 [11.8] and 28.6 [18.2]; *p* < 0.001 for grades I, II, III and IV, respectively) on the one hand and participants with grades I and IV, II and IV HOA (mean [SD]: 76.1 [12.9], 77.7 [19.9] and 41.5 [22.6]; *p* < 0.001 for grades I, II and IV, respectively) on the other hand for the SF-36 MC (Table 6).

## Discussion

The purpose of this study was to compare the impacts of the aetiology of HOA (primary and secondary) and radiographic status in three domains of the ICF model in Beninese participants with HOA. There was no significant difference between aetiology on structures and functions, activity and QoL in this study. However, the level of cartilage and bone degradation significantly impacted the participants on function, activity and QoL.

In this study, the average age was 40.5 (17.9) years. This suggests that HOA in the Republic of Benin affects young adult people. This may be attributed to the role of haemoglobinopathy in the occurrence of aseptic osteonecrosis of the femoral head, which was reported in 20 of the 49 participants. In aseptic osteonecrosis of the femoral head, bone cells or osteocytes are affected by a metabolic disorder and their nutrition modified by a simple local reduction in circulation below the threshold that ensures their survival (Lespasio et al. 2019; Narayanan et al. 2017). This local reduction in circulation is thought to result from ischemic thrombosis of the artery of the round ligament, the nourishing artery of the femoral head (Lespasio et al. 2019; Narayanan et al. 2017). This observation was reinforced by Oniankitan et al. (2009), who showed that epiphysis and aseptic osteonecrosis of the femoral head seem to constitute the main risk factors for secondary HOA in sub-Saharan Africa.

Participants were overweight with an average BMI of 25.6 (SD 7.4) kg/m^2^. Obesity can exert an increased load because of increased body weight; however, there may be differential systemic effects depending on the degree of fat versus lean mass. Body mass index may be associated with HOA in our participants. Indeed, an increasing load on a hip joint resulting from overweight may affect the appearance of HOA. This is consistent with the observations from Heliovaara et al. (1993) and Jiang et al. (2011), who reported a positive association between an increased BMI and HOA.

Participants with secondary HOA were not more severely affected, as no statistically significant differences were found for pain, ROM and strength. The young age profile of participants with HOA induced by aseptic osteonecrosis of the femoral head combined with lower BMI compared to participants with primary HOA could explain the lack of differences in scores. Future investigations should be envisaged to support our findings.

Radiographic state had an influence on the pain, activity and QoL of participants but not on hip joint ROM or muscle strength. Participants with grade IV HOA felt more pain. Pressure on the subchondral bones, sclerosis, and cysts observed in patients undergoing severe joint deterioration may be the explanation for their greater pain. This finding suggests that pain intensity may be partly related to the degree of HOA, but this assumption is only partially supported by research findings. Summers et al.’s (1988) study has shown that the degree of objective disease severity (ODS), a set of criteria for the radiographic assessment of disease severity in osteoarthritis (OA), was significantly correlated with a higher level of pain on the McGill pain questionnaire. Dougados et al. (1996) has suggested that there could be a statistically significant correlation between clinical and radiological parameters evaluating HOA. In a survey, Arokoski et al. (2004) showed that the pain score was not correlated with the degree of radiologic severity of OA. Veenhof et al.’s (2012) systematic review showed conflicting evidence in association between pain and greater joint degradation in HOA. The literature suggests that other factors, such as physical and psychosocial disability (Hopman-Rock et al. 1996), muscle weakness and ability to cope with pain (Van Baar et al. 1998) or duration of limitation in normal activities and level of education (Thumboo et al. 2002), are associated with the severity of pain in HOA. Other studies need to be conducted to support this finding.

There was no statistically significant difference according to the grades of HOA in the affected hip joint ROM and muscular strength in this study. This observation is consistent with what was reported in the existing literature indicating reduced hip joint ROM in a population with HOA (Arokoski et al. 2004; Rydevik et al. 2010; Steultjens et al. 2000). According to Arokoski et al. (2004), hip joint ROM is a sensitive marker of the radiographic severity of osteoarthritis. He showed that the worse the deterioration of the hip, the lower the ROM value of the hip detected in abduction and in internal and external rotations. More research is required to corroborate these results. However, this muscle deficiency observed in the participants may be associated with pain and limitation of activity as described in the literature (Rydevik et al. 2010; Van Baar et al. 1998).

In terms of activity, a statistically significant difference in the spontaneous gait speed was observed between participants with grades I and IV HOA. This difference may be attributed to a combination of many factors: (1) a greater BMI (mean 26 [SD 7.4) kg/m^2^]), more intense pain (NRS: median 7 [IQR 6–9]; OHS 19 [IQR 8.7–27]). It is therefore possible that the level of joint degradation of our participants and its consequences may lead to limitation of activity, resulting in a slow spontaneous gait. This slow spontaneous gait in participants, particularly in those with grade IV HOA, may be one strategy to counterbalance pain and joint load or joint deformities resulting from HOA severity. Several authors have reported the effects of HOA on activity but no strong association with radiographic state. For a few authors, there is low evidence for the association between level of physical activity and joint degradation (Veenhof et al. 2012), and radiographic OA correlated poorly with physical function (Thumboo et al. 2002).

Constantinou et al. (2017) found that patients with mild to moderate HOA walk slower and the walking distance was significantly shorter during the 6MWT (Rydevik et al. 2010). In a population survey, Odding et al. (1996) found that restricted ROM of several joint actions was associated with the presence of locomotor disability. According to Arokoski et al. (2004), people without HOA were significantly better at walking, ascending and descending stairs, and performing a 25-metre walk test. Future researches using more efficient tests need to be considered to confirm our observations.

The participants with grades I, II and III HOA had a better QoL than those with grade IV. Indeed, participants with grade IV HOA had more pain with a slower spontaneous gait than participants with grades I, II or III HOA. In accordance, Rydevik et al. (2010) reported that significantly lower health-related QoL in HOA patients is attributed to a reduction of hip ROM and an increase in joint stiffness. Arokoski et al. (2004) observed that the function and scores of WOMAC correlated (*p* < 0.05) with the degree of radiologically estimated severity of HOA. Some previous researches have reported that WOMAC is associated with the radiological severity of HOA (Bellamy 2005; Stucki et al. 1998).

## Strengths and limitations

To our knowledge, this is the first study to explore the influence of aetiology and the radiographic state of HOA on the structures and functions, the activity and the participation of a Beninese population with HOA. This is a first step that may help to establish better strategies for the management of this population (policy of identification of patients with HOA, an efficient platform for the best care of patients, etc.). The knowledge of HOA’s impact on the structures and function, activity and participation for a Beninese population with HOA may help clinicians to evaluate and develop rehabilitation programmes to improve functioning and reduce disability in this population.

However, this research has some limitations: (1) the sample size was small, and the recruitment was done exclusively in only one hospital reference centre; (2) other factors associated with HOA already studied in the literature were not taken into consideration; (3) the influence of environmental factors (home, work environment, social structures, communication and mobility services, laws and societal regulations, etc.) were not discussed in this study. Furthermore, the methodological choice to consider only the most painful hip in participants with bilateral HOA may constitute a bias. Further researches on the prevalence, association with other factors and treatment are needed and should be conducted on larger sample sizes. This will provide more information on the assessment and management of the population with HOA.

## Conclusion

The purpose of this study was to compare the impacts of aetiology and radiographic state in three domains of the ICF model in a Beninese population with HOA. The results showed that aetiologies did not impact the body structures and function, activity or participation. The severity of OA based on radiographic state showed significant impacts on these variables. Higher degradation led to more impacts on different domains of ICF.

## APPENDIX 1: Procedures used for the muscle test.

### Hip flexors

***Patient*:** Sitting upright, with the knees bent over the side of the table. Hold on to the table to prevent leaning backward to obtain assistance by two-joint hip flexors.

***Fixation*:** The weight of the trunk may be sufficient to stabilise the patient during this test, but holding on to the table gives added stability. If the trunk is weak, place the patient in the supine position during the test.

***Test*:** Hip flexion with the knee flexed, raising the thigh a few inches from the table.

***Pressure*:** Against the anterior thigh, in the direction of extension.

### Hip extensors

***Patient*:** Prone, with knee flexed 90° or more. (The more the knee is flexed, the less the hip will extend because of restricting tension of the rectus femoris anteriorly.)

***Fixation*:** Posteriorly, the back muscles; laterally, the lateral abdominal muscles; anteriorly, the opposite hip flexors fix the pelvis to the trunk.

***Test*:** Hip extension, with the knee flexed.

***Pressure*:** Against the lower part of the posterior thigh, in the direction of hip flexion.

### Adductors

***Patient*:** Lying on the right side to test the right (and vice versa), with the body in a straight line and the lower extremities and lumbar spine straight.

***Fixation*:** The examiner holds the upper leg in abduction. The patient should hold on to the table for stability.

***Test*:** Adduction of the underneath extremity upward from the table, without rotation, flexion or extension of the hip or tilting of the pelvis.

***Pressure*:** Against the medial aspect of the distal end of the thigh, in the direction of abduction (i.e. downward toward the table). Pressure is applied at a point above the knee to avoid strain of the tibial collateral ligament.

### Abductors

***Patient*:** Side-lying.

***Fixation*:** The examiner stabilises the pelvis.

***Test*:** Abduction of the hip in a position neutral between flexion and extension and neutral in regard to rotation.

***Pressure*:** Against the leg, in the direction of adduction, and very slight extension.

### Medial rotators

***Patient*:** Sitting on a table, with the knees bent over the side and the subject holding on to the table.

***Fixation*:** The weight of the trunk stabilises the patient during this test. Stabilisation is also given in the form of counterpressure, as described below under ‘Pressure’.

***Test*:** Medial rotation of the thigh, with the leg in a position of completion of the outward arc of motion.

***Pressure*:** With one hand, the examiner applies counterpressure at the medial side of the lower end of the thigh. With the other hand, the examiner applies pressure to the lateral side of the leg, above the ankle, pushing the leg inward in an effort to rotate the thigh laterally.

### Lateral rotators

***Patient*:** Sitting on a table, with the knees bent over the side and the subject holding on to the table.

***Fixation*:**The weight of the trunk stabilises the patient during this test. Stabilisation is also given in the form of counterpressure, as described below under ‘Pressure’.

***Test*:** Lateral rotation of the thigh, with the leg in a position of completion of the inward arc of motion.

***Pressure*:**With one hand, the examiner applies counterpressure at the lateral side of the lower end of the thigh. With the other hand, the examiner applies pressure to the medial side of the leg, above the ankle, pushing the leg outward in an effort to rotate the thigh medially.

## APPENDIX 3: Short-form 36-item health survey questionnaire, physical and mental components.

### Présentation:

Ce bilan de santé généraliste peut être utilisé en complément de bilans plus spécifiques.

### Critères d’inclusion (les catégories majeures cliniques):

Toutes catégories de personnes ayant des difficultés de santé.

### Critères d’exclusion (ne pas utiliser pour):

Aucun.

### Critères de péjoration (diagnostic associé):

Dépression, difficultés relationnelles.

### Evolution du score:

Varie selon les items, afin de tester la vigilance du patient. La lecture des résultats fournit une appréciation sémantique.

#### Le questionnaire généraliste SF-36

1. ***En général, diriez-vous que votre santé est:*** (cocher ce que vous ressentez)
  Excellente __ Très bonne __ Bonne __ Satisfaisante __ Mauvaise __
2. ***Par comparaison avec il y a un an, que diriez-vous sur votre santé aujourd’hui?***
  Bien meilleure qu’il y a un an __ Un peu meilleure qu’il y a un an __
  A peu près comme il y a un an __ Un peu moins bonne qu’il y a un an __
  Pire qu’il y a un an __
3. ***Vous pourriez vous livrer aux activités suivantes le même jour. Est-ce que votre état de santé vous impose des limites dans ces activités? Si oui, dans quelle mesure? (entourez la flèche).***
  - Activités intenses: courir, soulever des objets lourds, faire du sport.
    ____↓________________________↓_________________________↓____
    Oui, très limité Oui, plutôt limité Pas limité du tout
  - Activités modérées:déplacer une table, passer l’aspirateur.
    ____↓________________________↓__________________________↓____
    Oui, très limité Oui, plutôt limité Pas limité du tout
  - Soulever et transporter les achats d’alimentation.
    ____↓________________________↓_________________________↓____
    Oui, très limité Oui, plutôt limité Pas limité du tout
  - Monter plusieurs étages à la suite.
    ____↓________________________↓________________________↓____
    Oui, très limité Oui, plutôt limité Pas limité du tout
  - Monter un seul étage.
    ____↓________________________↓________________________↓____
    Oui, très limité Oui, plutôt limité Pas limité du tout
  - Vous agenouiller, vous accroupir ou vous pencher très bas.
    ____↓________________________↓________________________↓____
    Oui, très limité Oui, plutôt limité Pas limité du tout
  - Marcher plus d’un kilomètre et demi.
    ____↓________________________↓________________________↓____
    Oui, très limité Oui, plutôt limité Pas limité du tout
  - Marcher plus de 500 mètres
    ____↓________________________↓________________________↓____
    Oui, très limité Oui, plutôt limité Pas limité du tout
  - Marcher seulement 100 mètres.
    ____↓________________________↓________________________↓____
    Oui, très limité Oui, plutôt limité Pas limité du tout
  - Prendre un bain, une douche ou vous habiller.
    ____↓________________________↓________________________↓____
    Oui, très limité Oui, plutôt limité Pas limité du tout
4. ***Au cours des 4 dernières semaines, avez-vous eu l’une des difficultés suivantes au travail ou lors des activités courantes, du fait de votre santé? (réponse: oui ou non à chaque ligne)***
  **Table:**
  	oui	non
  Limiter le temps passé au travail, ou à d’autres activités?		
  Faire moins de choses que vous ne l’espériez?		
  Trouver des limites au type de travail ou d’activités possibles?		
  Arriver à tout faire, mais au prix d’un effort?		
5. ***Au cours des 4 dernières semaines, avez-vous eu des difficultés suivantes au travail ou lors des activités courantes parce que vous étiez déprimé ou anxieux? (réponse: oui ou non à chaque ligne).***
  **Table:**
  	oui	non
  Limiter le temps passé au travail, ou à d’autres activités?		
  Faire moins de choses que vous n’espériez?		
  Ces activités n’ont pas été accomplies aussi soigneusement que d’habitude?		
6. ***Au cours des 4 dernières semaines, dans quelle mesure est-ce que votre état physique ou mental ont perturbé vos relations avec la famille, les amis, les voisins ou d’autres groups?***
  ____↓_______________↓__________________↓___________________↓____
  Pas du tout Très peu Assez fortement Énormément
7. **Avez-vous enduré des souffrances physiques au cours des 4 dernières semaines?**
  ____↓_______________↓_________________↓____________________↓____
  Pas du tout Très peu Assez fortement Énormément
8. **- Au cours des 4 dernières semaines la douleur a-t-elle gêné votre travail ou vos activités usuelles?**
  ____↓_____________↓____________↓_______________↓___________________↓_____
  Pas du tout Un peu Modérément Assez fortement Énormément
9. ***- Ces 9 questions concernent ce qui s’est passé au cours de ces dernières 4 semaines. Pour chaque question, donnez la réponse qui se rapproche le plus de ce que vous avez ressenti. Comment vous sentiez-vous au cours de ces 4 semaines:***
  - vous sentiez-vous très enthousiaste?
    ____↓________________↓____________↓________________↓_______________↓___
    Tout le temps Très souvent Parfois peu souvent Jamais
  - étiez-vous très nerveux?
    ____↓________________↓____________↓________________↓_______________↓___
    Tout le temps Très souvent Parfois peu souvent Jamais
  - étiez-vous si triste que rien ne pouvait vous égayer?
    ____↓________________↓____________↓________________↓_______________↓___
    Tout le temps Très souvent Parfois peu souvent Jamais
  - vous sentiez-vous au calme, en paix?
    ____↓________________↓____________↓________________↓_______________↓___
    Tout le temps Très souvent Parfois peu souvent Jamais
  - aviez-vous beaucoup d’énergie?
    ____↓________________↓____________↓________________↓_______________↓___
    Tout le temps Très souvent Parfois peu souvent Jamais
  - étiez-vous triste et maussade?
    ____↓________________↓____________↓________________↓_______________↓___
    Tout le temps Très souvent Parfois peu souvent Jamais
  - aviez-vous l’impression d’être épuisé(e)?
    ____↓________________↓____________↓________________↓_______________↓___
    Tout le temps Très souvent Parfois peu souvent Jamais
  - étiez-vous quelqu’un d’heureux?
    ____↓________________↓____________↓________________↓_______________↓___
    Tout le temps Très souvent Parfois peu souvent Jamais
  - vous êtes-vous senti fatigué(e)?
    ____↓________________↓____________↓________________↓_______________↓___
    Tout le temps Très souvent Parfois peu souvent Jamais
10. ***Au cours des 4 dernières semaines, votre état physique ou mental a-t-il gêné vos activités sociales comme des visites aux amis, à la famille, etc.?***
  ____↓________________↓________________↓___________↓_______________↓___
  Tout le temps Très souvent Parfois Peu souvent Jamais
11. ***- Ces affirmations sont-elles vraies ou fausses dans votre cas?***
  - Il me semble que je tombe malade plus facilement que d’autres.
    ______↓____________↓_______________↓___________________↓_________↓_____
    Tout à fait vrai Assez vrai Ne sais pas Plutôt faux Faux
  - Ma santé est aussi bonne que celle des gens que je connais.
    ______↓____________↓_______________↓___________________↓_________↓_____
    Tout à fait vrai Assez vrai Ne sais pas Plutôt faux Faux
  - Je m’attends à ce que mon état de santé s’aggrave.
    ______↓____________↓_______________↓___________________↓_________↓_____
    Tout à fait vrai Assez vrai Ne sais pas Plutôt faux Faux
  - Mon état de santé est excellent.
    ______↓____________↓_______________↓___________________↓_________↓_____
    Tout à fait vrai Assez vrai Ne sais pas Plutôt faux Faux
    *Source:* Wade, J.E. & Sherbourne, C.D., 1992, ‘The MOS 36-item short-form health survey (SF-36)’, *Medical Care* 30, 473–483.

## APPENDIX 4: WOMAC: Index de sévérité symptomatique de l’arthrose des membres inférieurs.

Le WOMAC est l’index validé dans l’évaluation d’une arthrose des membres inférieurs. Il existe 2 systèmes de cotation des réponses aux questions: soit l’échelle de Likert avec 5 réponses possibles (nulle = 0 ; minime = 1 ; modérée = 2 ; sévère = 3 ; extrême = 4), soit une échelle visuelle analogique de 100 mm. Il est possible de calculer les scores dans chaque domaine ou pour l’ensemble du WOMAC

### WOMAC Domaine douleur: quelle est l’importance de la douleur?

(1) Lorsque vous marchez sur une surface plane? (2) Lorsque vous montez ou descendez les escaliers? (3) La nuit, lorsque vous êtes au lit? (4) Lorsque vous vous levez d’une chaise ou vous asseyez? (5) Lorsque vous vous tenez debout?

### WOMAC Domaine raideur

(1) Quelle est l’importance de la raideur de votre articulation lorsque vous vous levez le matin? (2) Quelle est l’importance de la raideur de votre articulation lorsque vous bougez après vous être assis, couché ou reposé durant la journée?

### WOMAC Domaine fonction: quelle est l’importance de la difficulté que vous éprouvez à:

(1) Descendre les escaliers? (2) Monter les escaliers? (3) Vous relever de la position assise? (4) Vous tenir debout? (5) Vous pencher en avant? (6) Marcher en terrain plat? (7) Entrer et sortir d’une voiture? (8) Faire vos courses? (9) Enfiler collants ou chaussettes? (10) Sortir du lit? (11) Enlever vos collants ou vos chaussettes? (12) Vous étendre sur le lit? (13) Entrer ou sortir d’une baignoire? (14) Vous asseoir? (15) Vous asseoir et vous relever des toilettes? (16) Faire le ménage ‘à fond’ de votre domicile? (17) Faire l’entretien quotidien de votre domicile?

Source: Bellamy, N., Buchanan, W.W., Goldsmith, C.H., Campbell, J. & Stit, L.W.J., 1995, ‘Validation of WOMAC: A health status instrument for measuring clinically important patient relevant outcomes to antirheumatic drug therapy in patients with osteoarthritis of the hip or knee’, *Journal of Rheumatology* 15(1), 1833–1840.

## APPENDIX 5: Consent form.

NOM ---------------------------PRENOM ----------------------Date Naissance --------------- DATE ----------------

### EVALUATION DE VOTRE PROBLEME DE SANTE SUR VOTRE VIE

#### Consentement éclairé

##### Participant

Je déclare que j’ai été informé sur la nature de l’étude, son but, et ce que l’on attend de moi. J’ai pris connaissance du document d’information.

J’ai eu le temps pour y réfléchir et en parler avec une personne de mon choix comme mon médecin généraliste ou un membre de ma famille.

J’ai eu l’occasion de poser toutes les questions qui me sont venues à l’esprit et j’ai obtenu une réponse satisfaisante à mes questions.

J’ai compris que ma participation à cette étude est volontaire et que les données me concernant seront récoltées pendant ma participation à cette étude et que le kinésithérapeute investigateur et le promoteur de l’étude se portent garant de la confidentialité de ces données.

Je consens au traitement de mes données personnelles selon les modalités décrites. Je donne également mon accord au transfert et au traitement de ces données dans d’autres pays que le Bénin.

J’accepte/n’accepte pas (biffer la mention inutile) que les données de recherche récoltées pour les objectifs de la présente étude puissent être traitées ultérieurement pour autant que ce traitement soit limité au contexte de la présente étude pour une meilleure connaissance de la maladie et de son traitement.

J’accepte/n’accepte pas (biffer la mention inutile) que mon médecin généraliste ou d’autres médecins spécialistes en charge de ma santé soient informés de ma participation à cette étude.

J’ai reçu une copie de l’information au participant et du consentement éclairé.

##### Nom, prénom, date et signature du volontaire.

____________________________________

##### Représentant légal

Je déclare que j’ai été informé qu’on me demande de prendre une décision de participation à l’étude de la personne que je représente au mieux de ses intérêts et en tenant compte de sa probable volonté. Mon consentement s’applique à tous les items repris dans le consentement du participant.

J’ai reçu une copie de l’information au participant et du consentement éclairé.

Nom, prénom et lien de parenté avec la personne représentée:

____________________________________

Date et signature du représentant légal.

#### Témoin/Interprète

J’ai été présent durant l’entièreté du processus d’information au patient et je confirme que l’information sur les objectifs et procédures de l’étude a été fournie de manière adéquate, que le participant (ou son représentant légal) a apparemment compris l’étude et que le consentement à participer à l’étude a été donné librement.

Nom, prénom et qualification du témoin/interprète:

____________________________________

Date et signature du témoin/interprète.

##### Kinésithérapeute Investigateur

Je soussigné, ------------------------------------------------------------------------ kinésithérapeute investigateur confirme avoir fourni oralement les informations nécessaires sur l’étude et avoir fourni un exemplaire du document d’information au participant.

Je confirme qu’aucune pression n’a été exercée pour que le patient accepte de participer à l’étude et que je suis prêt à répondre à toutes les questions supplémentaires, le cas échéant.

Je confirme travailler en accord avec les principes éthiques énoncés dans la dernière version de la « Déclaration d’Helsinki ».

Nom, prénom, Date et signature Nom, Prénom, Date et signature du représentant de l’investigateur du kiné investigateur

## APPENDIX 6: Hip range of motion based on grade of hip osteoarthritis in relation to norms

**Table:**

Variable	Grade I	Grade II	Grade III	Grade IV	Norms
Flexion (°)	108.82 (9.43)	103.43 (12.95)	101 (20.69)	91.6 (28.15)	150°
Extension (°)	20.6 (7.25)	19.57 (10.5)	19.54 (10.42)	19 (9.39)	30°
Abduction (°)	30 [20–44]	22 [20–40]	20 [17.5–34]	10 [10–32]	45–50°
Adduction (°)	20.7 (9.41)	18.83 (9.68)	18.91 (8.88)	18.47 (9.46)	20°
Internal rotation (°)	29.54 (10.03)	20.33 (12.09)	22.75 (10.06)	23.29 (6.72)	45°
External rotation (°)	30 [20–38]	19 [11.5–32.5]	16 [12–26]	20 [15.75–30.5]	45°

*Note:* Standard deviation is set in round brackets and interquartile range (IQR) is set in square brackets.
